# Use of SIICC / ACR damage index in adolescents with SLE

**DOI:** 10.1186/1546-0096-12-S1-P329

**Published:** 2014-09-17

**Authors:** Ludmila Bogmat, Nataly Shevchenko, Elena Matvienko

**Affiliations:** 1 Department of Cardiorheumatology, Institute of Children and Adolescents Health Care, Kharkiv, Ukraine

## Introduction

System Lupus Erythematoыus (SLE) is one of the most severe and unfavorable to its course of rheumatic diseases. Poor prognosis of SLE development has irreversible changes of internal organs. The number and type of there have accounted for by SIICC/ACR Damage Index in adults. However, such changes have found in children also.

## Objectives

In order to timely diagnosis of irreversible changes of internal organs in systemic lupus erythematosus (SLE) 44 adolescents with SLE medical history have analyzed.

## Methods

The age of patients at the time of the study were 12,67 ± 3,17 years. SLE debut age equaled 12,65 ± 2,87 years. Duration of illness was 39,36 ± 4,17 months.

The diagnosis of SLE exhibited respectively ACR classification criteria (1997). SIICC /ACR Damage Index was assessed in all patients. Comparison of estimates of the index had conducted at duration of illness before and more than three years.

## Results

SLE manifested the following clinical syndromes: articular (84.3%), skin (82.3%), renal (55.4%), cardial (59.8%), pulmonary (35.7%), cerebral (31, 2%), Blood disorders (64.3%), antiphospholipid syndrome (11.9%).

Most patients had subacute start option (61.4%) and moderate activity of SLE (45.41%).

All adolescents were treated with glucocorticoids; the mean total dose was 11326 ± 2435 mg per patient. Combination therapy with glucocorticoids and cytotoxic drugs was at 70.3% of patients. Ultrahigh doses of methylprednisolone (“pulse”- therapy) have conducted in 37.5% of patients.

The authors have found that 90.9% of adolescents with SLE have the damage that includes the scale SIICC /ACR Damage Index for adult patients. Average score SIICC / ACR Damage Index in adolescents with SLE was 2,72 ± 0,84 points. Frequency of cumulative damage and the total score SIICC/ACR Damage Index increased with disease duration of more than three years. The structure damage had the greatest increase of cardiovascular changes (21.2% vs. 12.2%), neuropsychiatric disorders (12.1% vs. 3.0%), renal disease (3.0% vs 0.9% ). Aseptic necrosis of bone tissue occurred when the duration of the disease for more than three years only (3.1%).

## Conclusion

Research proves that children with SLE should be have evaluated to assess the scale of damage. Adaptation of these scales have needed for children and adolescents.

## Disclosure of interest

None declared.

